# Current concepts and recent advances in understanding and managing lumbar spine stenosis

**DOI:** 10.12688/f1000research.16082.1

**Published:** 2019-01-31

**Authors:** Carlos Bagley, Matthew MacAllister, Luke Dosselman, Jessica Moreno, Salah G. Aoun, Tarek Y. El Ahmadieh

**Affiliations:** 1 Neurosurgery, University of Texas Southwestern Medical Center at Dallas, Dallas, TX, USA

**Keywords:** lumbar stenosis, neurogenic claudication, lumbar radiculopathy

## Abstract

Lumbar spinal stenosis is a degenerative process that is extremely frequent in today’s aging population. It can result in impingement on the nerves of the cauda equina or on the thecal sac itself, and lead to debilitating symptoms such as severe leg pain, or restriction in the perimeter of ambulation, both resulting in dependency in daily activities. The impact of the disease is global and includes financial repercussions because of its involvement in the active work force group. Risk factors for the disease include some comorbidities such as obesity or smoking, daily habits such as an active lifestyle, but also genetic factors that are not completely elucidated yet. The diagnosis of lumbar stenosis can be difficult, and involves a combination of radiological and clinical findings. Treatment ranges from conservative measures with physical therapy and core strengthening, to steroid injections in the facet joints or epidural space, to a more radical solution with surgical decompression. The evidence available in the literature regarding the causes, diagnosis and treatment of lumbar spine stenosis can be confusing, as no level I recommendations can be provided yet based on current data. The aim of this manuscript is to provide a comprehensive and updated summary to the reader addressing the multiple aspects of this disease.

## Introduction

Lumbar spinal stenosis (LSS) is a pathological process where bony, ligamentous, and synovial elements of the lower axial spine degenerate and overgrow, progressively compressing the neural and vascular elements in the spinal canal. This degenerative process may result in impingement on the nerve roots of the cauda equina ^1^. Congenital factors may predispose some individuals to this condition ^1^. The compression can be asymptomatic if it is mild or can result in a variable combination of static back pain, radicular lower extremity pain, or neurogenic claudication. Static symptoms are typically exacerbated by ambulation or extension of the lumbar spine and are temporarily relieved by sitting or lumbar flexion maneuvers, thus promoting an increasingly kyphotic posture in patients with the disease ^1, 2^.

The diagnosis of LSS is made by reconciling an array of clinical symptoms with radiographic findings of lumbar stenosis on computed tomography (CT) or magnetic resonance imaging (MRI). The prevalence of LSS increases with age ^1^, and population-based radiographic studies of adults over the age of 40 have estimated the prevalence of moderate stenosis to range anywhere from 23.6 to 77.9%; severe stenosis occurs in 8.4 to 30.4% of individuals ^1, 2^. However, the percentage of the population experiencing clinical symptoms appears to be significantly smaller. In a population-based cohort study of 1009 Japanese subjects, Ishimoto *et al*. estimated that approximately 9.3% of adults have symptomatic LSS and that the prevalence is greater in men than in women ^3^. The most important risk factor for LSS is age, and additional factors include obesity, congenital spinal stenosis, tobacco use, and occupational hazard with repetitive spinal stress, which are all elements predisposing to chronic lower back pain ^4^. Patients with LSS can experience debilitating pain and often have weakness, which makes ambulation and the performance of normal activities of daily living difficult. This can result in an increasingly sedentary and dependent lifestyle, which further perpetuates the condition. This often leads to losses in both productivity and quality of life, which is why LSS is one of the most common indications for lumbar spine surgery ^5^. In 2007, more than 37,500 operations for spinal stenosis were performed in Medicare patients in the United States alone, and the total cost was almost $1.65 billion ^5^. The purpose of this article is to review the pathophysiology, diagnosis, and clinical course of this common and potentially debilitating disease. We will also provide a brief summary of the current state of evidence for the different surgical and medical therapeutic options available.

## Pathophysiology

LSS can be congenital or acquired, and the degenerative acquired form is by far the most common ^1^. Acquired stenosis is thought to result from a cascade of changes initiated by the degeneration of the nucleus pulposus of the intervertebral disc as patients age. Degeneration and atrophy of stabilizing axial musculature, repeated trauma to the axial spine from daily wear and tear, and potential occupational hazards lead to the desiccation of the nucleus and collapse of the disc space. This process may be exacerbated by weak or degenerating axial musculature, especially if coupled with fatty infiltration of the paraspinal girdle, and excessive body weight ^6^. The collapse of the disc space destabilizes and shortens the anterior spinal column, shifting axial stress toward the posterior elements, including the facet joints, the interspinous ligament, the ligamentum flavum, and the subarticular ligaments ^7^. This chronic excessive stress results in joint overgrowth with synovial hypertrophy, synovial cyst and osteophyte formation, and posterior ligamentous buckling and thickening. These factors combine to cause the narrowing of the spinal canal. The narrowing results anteriorly from disc collapse and herniation, laterally from facet and subarticular ligament overgrowth, and posteriorly from ligamentum flavum buckling and thickening ^8^. Degenerative spondylolisthesis (DS), which consists of the anterior displacement of a vertebral body over the disc space, with or without bony pars defect, can also be an important contributor to spinal stenosis ^9, 10^. It is now evident that, in addition to acquired degeneration that can promote LSS, genetic factors play a big role in bony canal stenosis as well as thecal sac size and can have a significant impact on clinical outcome ^11^. This genetic predisposition may account for the variation in population-based prevalence estimates of moderate (24% versus 78%) and severe (8% versus 30%) stenosis in adults over the age of 40 ^11^.

LSS can be preferentially central, lateral, or foraminal ^7^. Central stenosis is associated mainly with axial back pain and neurogenic claudication, and motor or sensory radicular symptoms are possible. The pain associated with central canal stenosis is typically bilateral, and the lumbar levels most commonly involved are L4–5 level followed by L3–4 and L5–S1 ^12^. There are two theories explaining the mechanism by which central stenosis results in neurogenic claudication pain. The ischemic theory postulates that compression causes decreased arterial flow to the nerve roots, generating ischemic pain and weakness. The venous stasis theory, on the other hand, supposes that venous blood stasis leads to inadequate oxygenation of the capillary bed, the accumulation of metabolites in the cauda equina, and subsequent pain and claudication ^13, 14^.

Lateral recess and foraminal stenosis may be unilateral and cause impingement of the traversing root or the exiting root at the subarticular recess and the foramen, respectively ^12^. In lateral recess stenosis, the traversing segment of the nerve root is compressed by the facet joint and subarticular ligament hypertrophy. Foraminal stenosis can be caused by scoliosis, lateral or foraminal disc, or synovial cyst and can impact the nerve or the sensory ganglion ^12, 15, 16^. Foraminal stenosis results in a unilateral radiculopathy with pain and possibly weakness of the corresponding muscular territory.

## Diagnosis

The diagnosis of LSS is usually made through the association of clinical symptoms with the presence of lumbar canal stenosis with thecal sac impingement on imaging ^7^. A systematic review by de Schepper *et al*. found that the most sensitive clinical finding is radiating leg pain that is exacerbated while standing ^17^. Bilateral buttock or leg pain that resolves when sitting or when bending forward and a wide-based gait were also found to be reasonably sensitive and specific symptoms. More clearly defined physical examination signs such as a positive straight leg raise test were found to have a lower diagnostic value. The International Delphi Study proposed a set of seven history items that, if positive, would help clinicians define LSS with improved accuracy both in the clinical and in the research setting ^18^. Differentiating neurogenic from vascular claudication is also important, and the former improves with anterior kyphotic flexion and sitting whereas the latter improves with resting of the affected limb.

A peripheral vascular exam often reveals absent dorsalis pedis or tibialis anterior pulses with a positive Buerger’s test in vasculopathic claudicators. If the clinician is in doubt, arterial imaging should be performed and occasionally vascular and neurogenic claudication may be co-existent.

MRI is generally preferred as a diagnostic imaging tool because of its superior soft tissue resolution. If MRI is contraindicated, CT with myelography has a similar diagnostic capacity in detection of the stenosis but with the caveat that it is invasive and ionizing and cannot reveal intrinsic spinal pathology. Plain non-contrasted CT scans still have their uses as they allow us to detect ligamentous and disc space calcification, which can influence the surgical plan. Plain film is also very useful and is the only commonly used imaging study that showcases the patient’s anatomy in a position of axial loading and reflects the patient’s biomechanical balance in the standing or sitting positions. Dynamic x-rays (flexion and extension) can be helpful in determining whether the patient has dynamic instability and thus would require a fusion procedure in addition to the lumbar decompression. Although there are MRI or CT machines where the patient is scanned in a reclined or standing position, they are not yet a routine part of standard clinical practice and, if available, can be organized if supine MRI scans are not diagnostic of significant stenosis but there is high clinical suspicion ^19– 21^.

Defining LSS radiographically remains challenging because of the lack of formally standardized radiologic criteria. The lower limit of a normal antero-posterior diameter for the lumbar spinal canal has been established as 15 mm on plain lateral radiographs, and congenital stenosis is defined as less than 10 mm ^22, 23^. In the CT era, the cross-sectional area (CSA) of the thecal sac itself has become the measurement of reference. In cadaveric studies evaluating pressure changes with compression of the cauda equina, Schonstrom *et al*. defined thecal sac CSA values of less than 75 mm ^2^ and less than 100 mm ^2^ for absolute and relative LSS, respectively ^23, 24^. These thresholds are still widely accepted and used in clinical studies today ^23^. Several additional indices and grading systems have been created ^25, 26^ but either are too cumbersome to compute or do not correlate well with the clinical manifestation of the disease. Overall, there is poor correlation between stenosis on imaging and a patient’s clinical symptoms, and imaging alone is not sufficient to make a diagnosis but has to be correlated with the patient’s symptoms and history ^27^.

Electrodiagnostic testing (electromyography and nerve conduction studies) is not routinely recommended for patients with suspected LSS. However, in individuals with an atypical presentation, inconclusive imaging, or concern for confounding etiologies, such as lumbar plexopathies, nerve impingement syndromes, vascular claudication, or metabolic neuropathies, these tests may be useful when combined with a good clinical exam ^28^.

## Clinical course

Reliable evidence describing the natural history of LSS is lacking for severely advanced cases because most patients seek treatment and because surgical treatment is usually well tolerated with resolution of claudication and radicular symptoms. On the other hand, the North American Spine Society estimates that the natural history of patients with mildly to moderately symptomatic lumbar stenosis can be favorable in up to 50% of patients and that a rapid or catastrophic neurologic decline is rare ^19^. A 4-year study of patients with untreated LSS found that symptoms were unchanged in 70%, improved in 15%, and worsened in 15% of cases. In a similar 10-year study of a cohort of 34 patients who presented with LSS and received conservative treatment, symptoms improved in about 30%, remained unchanged in 30%, and worsened in 30% of cases ^29^.

## Treatment options

### Medical treatment

The treatment options of LSS vary widely in clinical practice and may include medication, bracing, exercise and physical therapy, transcutaneous electrical nerve stimulation (TENS), epidural steroid injections (ESIs), or surgical decompression. The choice of therapy is usually made by combining clinical evidence of recommendation with individual patient characteristics and patient preference. Surgical decompression is generally reserved for patients with moderate to severe symptoms that have had a chronically worsening course or failed an initial line of conservative measures. Unfortunately, there is still no high-quality evidence regarding conservative management options. A systematic review published in 2013 concluded that the current evidence was not sufficient to provide official guidelines for clinical practice ^30^. However, some patients may experience both short- and long-term symptomatic relief with conservative treatment and expert consensus ^19^.

Medication is prescribed primarily for symptomatic pain relief, usually to enable the patient to go through the initial phase of physical therapy and not as a long-term measure. Non-steroidal anti-inflammatory drugs (NSAIDs) are the most commonly recommended first-line agents. There is no evidence to support the use of one type of NSAID over another, nor is there conclusive evidence that acetaminophen may be clinically effective ^31, 32^. Opioids and muscle relaxants have not been shown to be superior to NSAIDs or acetaminophen. Other agents such as prostaglandins, gabapentin, vitamin B _12_, and calcitonin have been studied but their role remains unclear at this time ^13^.

### Epidural injections

ESIs are viewed as a relatively safe and less invasive alternative to surgical intervention. The 2013 North American Spine Society committee guidelines suggested the use of ESI to provide short-term symptom relief in patients with neurogenic claudication or radiculopathy ^19^. However, a more recent systematic review (published in 2015) concluded that ESI offered minimal to no symptomatic relief or improvement in the walking ability in patients with LSS ^33^. In regard to the type of medication injected, a large double-blind multi-site trial published in 2014 found that the ESI of glucocorticoids plus lidocaine offered minimal or no short-term benefit compared with epidural injection of lidocaine alone ^34^.

### Physical therapy

Physical therapy for LSS usually involves some combination of core strengthening, flexibility training, and stability exercises. The optimal combination of these exercises and their frequency, duration, and appropriate setting is not clear at this time ^30^. Low-quality evidence from small studies suggests that any form of rehabilitation may result in improvements in pain and function ^30^. The evidence of benefit from physical therapy alone is not clear. However, a limited course of physical therapy should still be considered as part of the initial treatment discussion and conservative measures. Another important role for physical therapy, which is supported by moderate-quality evidence, is in the period following spine surgery. A systematic review of randomized controlled trials (RCTs) published in 2014 found that active rehabilitation initiated six weeks to three months after surgery is more effective than standard care for long-term improvement in functional status, low back pain, and leg pain ^35^. A multi-center RCT by Delitto *et al*. comparing physical therapy with surgery in patients who were all potential surgical candidates found physical therapy to potentially be as efficient as surgery, although these results should be critically analyzed given the number of patients who crossed over to the surgical arm and the intention-to-treat analysis of the study ^36^.

### Other conservative measures

Semi-rigid lumbosacral bracing has been found to potentially improve the walking perimeter and decrease pain in a minority of patients ^19^. Additional conservative therapy options such as TENS units, acupuncture, or spinal manipulation have not been sufficiently studied in robust clinical trials to provide clinically supported recommendations ^19^.

### Surgical management

For patients who do not improve with conservative management or who have severe symptoms and thecal sac compression at presentation, surgical intervention is generally recommended. A systematic review of the literature has shown that delaying surgery for a period of conservative management is not associated with a worse surgical outcome and that surgery is more effective than continued conservative treatment when conservative options have failed for a period of three to six months ^37^. There are many different surgical approaches for treating LSS, which include open, minimally invasive, and endoscopic procedures. At this time, no evidence-based guidelines are available for determining whether a specific approach should be used in certain scenarios or in a specific category of patients. The best surgical option should be determined on the basis of the anatomic location of the stenosis, the number of levels involved, the involvement of the thoracolumbar junction, and the presence of transitional anatomy or of instability or deformity. The goal of each approach is to decompress the compromised neural elements and provide symptomatic relief while preventing further degeneration in a way that does not destabilize the spine.

Several randomized trials have been performed to compare the effectiveness of surgical decompression with conservative management. The Spine Patient Outcomes Research Trial (SPORT) is the largest study that compared standard posterior decompressive laminectomy with non-operative management in patients with LSS without spondylolisthesis. The analysis of this study was somewhat clouded by a high crossover rate; however, in the intention-to-treat analysis, there was a significant treatment effect favoring surgery for reduction in pain. In addition, in the as-treated analysis, patients who were treated surgically had substantially greater improvement in pain and function at two years ^38^. At four years, the authors published additional follow-up data which demonstrated sustained improvement in pain and function in favor of surgery ^39^.

The role of spinal fusion in addition to decompression remains controversial at this time. In the 1990s, two small trials showed that patients with LSS with DS had better outcomes when fusion was performed at the time of the laminectomy ^40, 41^. Subsequently, laminectomy and fusion became the standard practice for LSS with DS, and rates of lumbar fusion surgery increased significantly ^42^. A large cohort study (5390 patients) published in 2013, however, showed no difference in patient satisfaction with the addition of fusion versus decompression alone ^43^. In 2016, two RCTs were published with conflicting results. The Swedish Spinal Stenosis Study, a large RCT comparing decompression plus fusion versus decompression alone, found no significant difference in clinical outcome or reoperation rates between the two groups at two and five years of follow-up regardless of the presence of DS ^44^. Similar results were found in a multinational registry study involving three Scandinavian countries ^45^. However, the Spinal Laminectomy **versus** Instrumented Pedicle screw (SLIP) study, an RCT looking specifically at patients with DS and LSS, found an improvement in physical health-related quality of life and lower rates of reoperation in the patients treated with decompression plus fusion versus decompression alone ^46^. Both studies found that fusion surgery was associated with higher cost, more blood loss, and longer hospital stays ^44, 46^.

## Surgical technique

Surgical decompression is traditionally achieved through an open bilateral laminectomy. However, minimally invasive surgical approaches that preserve stabilizing paraspinal musculature have recently become popular. These techniques seek to achieve bilateral laminar decompression typically through a smaller unilateral muscular incision with the use of a tubular retractor and microscope or endoscope ^47, 48^. A systematic review of the literature and meta-analysis which included five RCTs and seven observational studies comparing minimally invasive versus open laminectomy for LSS was published in 2016. The authors found that minimally invasive decompression was associated with higher satisfaction and lower pain scores with similar complication rates, lower blood loss, and shorter hospital length of stay but significantly longer operation time ^49^. A Norwegian spine registry analysis by Nerland *et al*. showed equivalent outcomes at one year between micro-decompression and laminectomy ^50^. Another minimally invasive surgical option for LSS is the insertion of interspinous process devices. These devices aim to indirectly decompress the neural elements by increasing the space between adjacent spinous processes simulating the flexion that often relieves symptoms for patients with LSS. They may be used as a stand-alone intervention or in combination with surgical decompression. RCTs comparing interspinous process devices with standard conventional surgical decompression have shown comparable outcomes for symptomatic relief but also much higher rates of reoperation associated with their use ^51, 52^.

## Predictors of outcomes after surgery

Several patient factors have been identified to predict outcomes after surgery for LSS. Age and gender do not appear to have a significant impact on prognosis after surgery ^53, 54^. Patients with preoperative depression tend to have worse outcomes than those without mood disorders ^55^. Preoperative optimization should include assessment and treatment of depression. Smokers and obese patients have also been found to have suboptimal improvement with surgery compared with non-smoking and normal-weight individuals ^56– 58^, indicating that smoking cessation and weight loss may also be important for preoperative counseling. Additionally, patients with predominant leg pain symptoms have been shown to improve significantly more with surgery than patients with predominantly lower back pain, although both groups still show improvement. This knowledge may be important in setting appropriate patient expectations before surgery ^59, 60^. Predictors for worsening following surgery have also been researched and may include young age and smoking ^61^.

## Future directions

Given the physical, psychological, and quality-of-life implications of LSS and its treatment, a multidisciplinary approach to management of this complex disorder should be undertaken. A team consisting of surgeons, physical therapists, interventionalists, and psychologists is necessary in order to determine the right treatment for the right patient at the right stage of their disease course. The role of increasingly popular “non-traditional” treatment options must be studied in order for clinician and patients to understand where these techniques may fit in the overall armamentarium of options. In addition, robust capture and analysis of patient outcomes at all phases of care and for the various treatment types are necessary on both the local and national levels in order to better assess the efficacy of the array of treatment options available for patients.

There is growing interest in motion preservation surgery in lieu of spinal fusion in the surgical community. Although this may be an attractive option for young patients with minimal degeneration, future advances may enable motion preservation in the older population presenting with degenerative LSS. Stem cell research has shown some anecdotal promise in improving fusion rates ^62^ but may play a role in regenerative treatments for degenerative LSS in the future ^63– 65^. Machine learning-based analysis could also be useful in creating prediction algorithms for good outcome after surgery and has the potential of efficiently analyzing large data repositories that are currently too complex to study thoroughly ^66^.
